# Distribution of interneurons in the CA2 region of the rat hippocampus

**DOI:** 10.3389/fnana.2014.00104

**Published:** 2014-09-26

**Authors:** Nicola A. Botcher, Joanne E. Falck, Alex M. Thomson, Audrey Mercer

**Affiliations:** Department of Pharmacology, University College London School of PharmacyLondon, UK

**Keywords:** interneurons, parvalbumin, CCK, calbindin, somatostatin, reelin

## Abstract

The CA2 region of the mammalian hippocampus is a unique region with its own distinctive properties, inputs and pathologies. Disruption of inhibitory circuits in this region appears to be linked with the pathology of specific psychiatric disorders, promoting interest in its local circuitry, its role in hippocampal function and its dysfunction in disease. In previous studies, CA2 interneurons, including a novel subclass of CA2 dendrite-preferring interneurons that has not been identified in other CA regions, have been shown to display physiological, synaptic and morphological properties unique to this sub-field and may therefore play a crucial role in the hippocampal circuitry. The distributions of immuno-labeled interneurons in dorsal CA2 were studied and compared with those of interneurons in CA1 and CA3. Like those in CA1 and CA3, the somata of CA2 parvalbumin-immunoperoxidase-labeled interneurons were located primarily in *Stratum Pyramidale* (SP) and *Stratum Oriens* (SO), with very few cells in *Stratum Radiatum* (SR) and none in *Stratum Lacunosum Moleculare* (SLM). There was, however, a greater proportion of GAD-positive cells were immunopositive for PV in SP in CA2 than in CA1 or CA3. CA2 SP also contained a larger density of somatostatin-, calbindin-, and VIP-immunopositive somata than CA1 and/or CA3. Like those in CA1 and CA3, CCK-immunopositive somata in CA2 were mostly located in SR. Reelin- and NPY- immunolabeled cell bodies were located in all layers of the three CA regions. However, a higher density of Reelin-positive somata was found in SP and SR of CA2 than in CA1 or CA3.

## Introduction

The intrinsic hippocampal circuitry was for many years considered to involve three main functional subregions: the dentate gyrus, the CA3 and the CA1 regions. Increasing evidence, however, suggests that the CA2 region is a distinctive subregion that may play a unique role in social memory (Hitti and Siegelbaum, 2014). CA2 pyramidal cells receive Schaffer collaterals inputs (Chevaleyre and Siegelbaum, 2010; Jones and McHugh, 2011), direct input from the entorhinal cortex (Bartesaghi and Gessi, 2004; Bartesaghi et al., 2006; Ding et al., 2010), the amygdala (Berretta et al., 2001), the dentate gyrus (Kohara et al., 2014) and uniquely amongst CA regions, inputs from the supramammillary nucleus, a hypothalamic nucleus thought to be involved in the generation of theta rhythms (Magloczky et al., 1994; Piskorowski and Chevaleyre, 2011; Cui et al., 2013). CA2 has also been shown to project directly to Layer II of the medial entorhinal cortex (MEC) (Rowland et al., 2013). Typically, the morphology of CA2 pyramidal cells was found to be similar to that described in CA1 (Mercer et al., 2007). However, interneurons in this region displayed different features. To date, more than 20 classes of interneurons have been reported in CA1 and CA3 (Somogyi and Klausberger, 2005; Ascoli et al., 2008; Fuentealba et al., 2008; Klausberger, 2009). They are generally identified on the basis of their dendritic and axonal arborisations, and the post-synaptic domain of the pyramidal cell that they target (Han et al., 1993; Sik et al., 1995; Buhl et al., 1996). In addition, they can be characterized according to their calcium binding protein (Parvalbumin [PV], Calbindin [CB], Calretinin [CR]) or neuropeptide content expression (Cholecystokinin [CCK], Somatostatin [SOM], Vasoactive Intestinal polypeptide [VIP], Neuropeptide Y [NPY]) (Somogyi and Klausberger, 2005; Ascoli et al., 2008; Fuentealba et al., 2008; Klausberger, 2009). So far, only three types of interneurons have been characterized morphologically and physiologically in the CA2 region: basket cells, bistratified cells and SP-SR interneurons. Some of these interneurons demonstrated striking differences from their equivalent in CA1 (Mercer et al., 2007, 2012a,b). Some CA2 PV-immunopositive basket and bistratified cells had partly spiny horizontal dendrites that extended horizontally along *stratum oriens* (SO) into both CA1 and CA3. These neurons displayed spike frequency adaptation and a “sag,” in response to hyperpolarizing current injection. These characteristics are strikingly different from those of CA1 PV- and CCK-basket cells, but resemble those of the OLM cells of the CA1 region and horizontally oriented CCK-immunopositive interneurons near the *stratum radiatum* (SR)*/stratum lacunosum moleculare* (SLM) border, Schaffer associated cells (Pawelzik et al., 2002). CA2 basket cell axons arborized in *stratum pyramidale* (SP) of all three CA regions, while the axons of CA2 bistratified cells were very strongly polarized, ramifying in SO and SR of the CA2 and CA1 regions but stopping abruptly at the CA2/CA3 border. The strength and time course of synaptic depression and facilitation were found to correlate with the class of interneuron. Basket and bistratified cells with narrow arbors, like those in CA1, received EPSPs that displayed pronounced depression, while EPSPs elicited in CA2 basket cells with broad dendritic arbor exhibited facilitation and limited augmentation. Another study also revealed a subclass of dendrite-preferring interneurons in CA2 that had not been previously described in any CA regions, CA2 SP-SR interneurons (Mercer et al., 2012b). The major difference between these interneurons and others classes was the region-specificity of their axonal arbors which exclusively innervated SR and their striking electrophysiological and physiological features. These neurons were PV- and CCK-immunonegative. CA2 SP-SR interneurons may play a key role in the hippocampal circuitry receiving inputs from all three CA regions and from Layer II of the entorhinal cortex and inhibiting CA2 pyramidal cells as well as neighboring CA1 and CA3 pyramidal cells.

Dysfunction of hippocampal circuitry and in particular of GABAergic systems in this particular small region appear to be contributory factors in some psychiatric diseases. The uniqueness of the three previously described types of interneurons in the CA2 region suggests that there may be other subtypes specific to this region. In the present study, immunoperoxidase staining was used to study the distributions of CA2 interneurons according to their calcium binding protein, peptide or protein content and to compare these distributions with those of interneurons in the CA1 and CA3 regions of the dorsal hippocampus, a part of the hippocampus that formed the basis of the majority of previous studies on the CA1 and CA3 regions.

## Methods

### Immunohistochemistry

**Figure 1:**
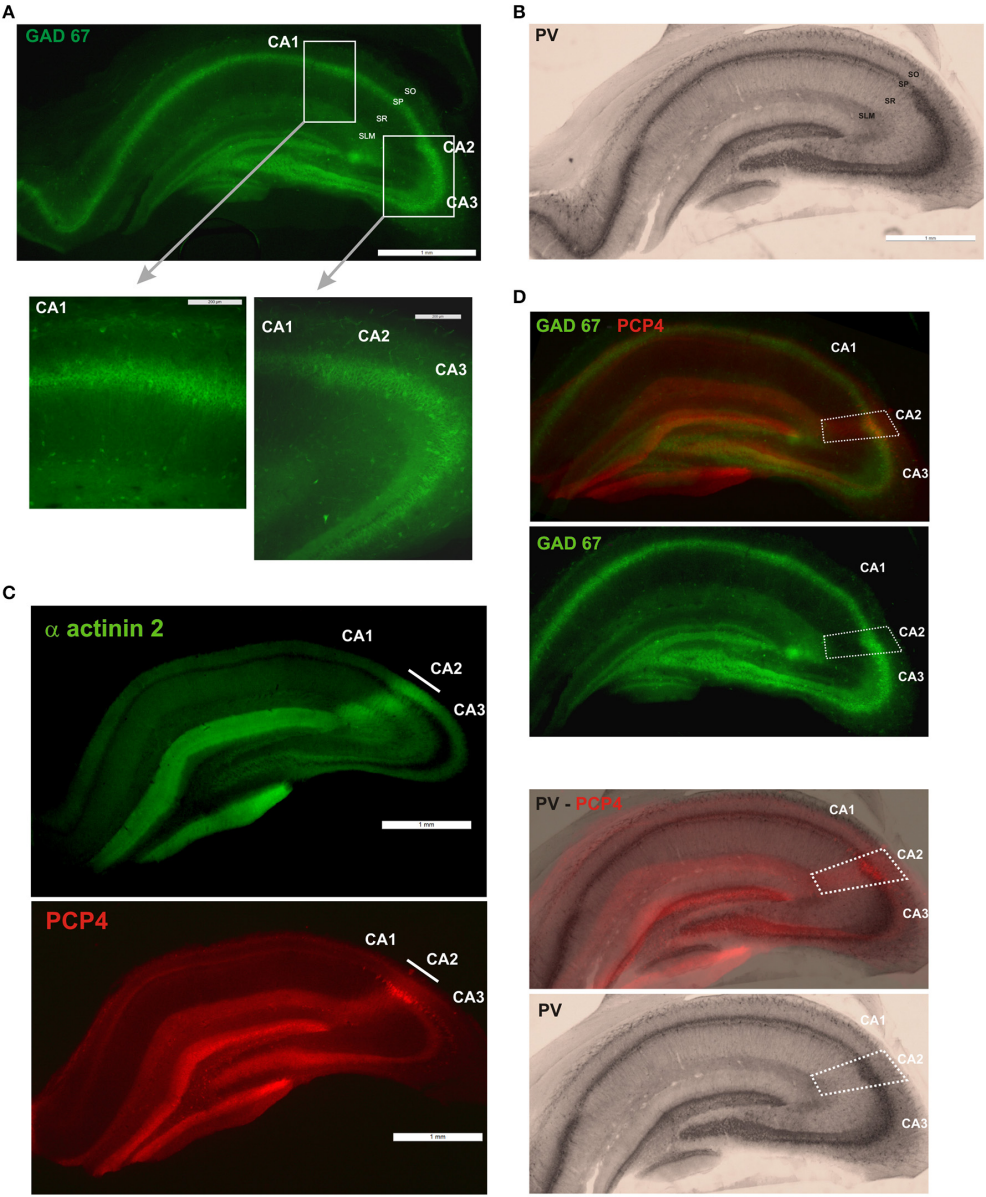
**Definition of the borders of the CA2 region. (A)** GAD67 fluorescent staining (scale bar 1 mm). GAD-positive neurons in the CA1, CA2, and CA3 regions are shown at higher magnification in the bottom panels (scale bars 200 μm). SO, Stratum Oriens; SP, stratum pyramidale; SR, stratum Radiatum; SLM, Stratum Lacunosum Moleculare; SL, Stratum Lucidum. **(B)** Immunoperoxidase staining of parvalbumin (PV)-positive interneurons (scale bar 1 mm). **(C)** Immunofluorescence staining for α actinin 2 protein and purkinje cell protein 4 (PCP4) determined the borders of the CA2 region. Scale bars, 1 mm. **(D)** The borders of CA2 (white dotted line) were defined by superimposition of the PCP4 labeling and GAD67 and immunoperoxidase stainings.

All procedures used throughout this study were carried out according to the British Home Office regulations with regard to the Animal Scientific Procedures Act 1986. Young adult male Wistar rats weighing 130–160 g were anaesthetized with inhaled Fluothane and then by intra-peritoneal injection of Euthatal (Merial, Harlow, UK) (60 mg/kg). The rats were perfused transcardially with ice cold oxygenated modified artificial cerebrospinal fluid (mACSF) containing in mM: 248 Sucrose, 25.5 NaHCO_3_, 3.3 KCl, 1.2 KH_2_PO_4_, 1 MgSO_4_, 2.5 CaCl_2_, 15 mM D-Glucose equilibrated with 95% O_2_/5% CO_2_. Brain were removed, and 500 μm thick slices containing dorsal hippocampus were cut with a vibratome (Vibroslice, Camden Instrument, Loughborough, UK) and fixed overnight (4% paraformaldehyde, 0.2% saturated picric acid solution, 0.025% glutaraldehyde solution in 0.1 M Phosphate buffer). Slices were washed, gelatin-embedded and 50 μm coronal sections cut. Sections were cryoprotected with sucrose and freeze-thawed. Sections were then incubated in 1% Sodium Borohydride (NaBH_4_) for 30 min and in 10% normal goat serum (NGS) for another 30 min. Sections were incubated overnight at 4°C in a primary antibody solution (mouse anti-GAD 67; MAB5406; 1:10,000, Millipore). Sections were then incubated in a secondary antibody solution, anti-mouse fluorescein isothiocyanate (FITC), made up in PBS. Sections were mounted on slides in Vectashield (Vector Laboratories) and studied by fluorescence microscopy as shown in Figure 1A.

Sections were then washed and incubated overnight at 4°C in a second primary antibody solution made up in PB. The list of primary antibodies used is presented in Table 1. The specificity of each antibody is detailed below. The same sections were then washed and incubated overnight in a secondary antibody solution, biotinylated goat anti-mouse or anti-rabbit antibody (1:200, Vector Laboratories) made up in PBS. To visualize the stained interneurons, sections were incubated first in ABC for 2 h and then in DAB (3, 3′ diaminobenzidine, Sigma). The sections were placed onto Fisher Superfrost slides, dehydrated and mounted (RAM mounting medium, VWR). Only the sections that were intact following both the GAD67 staining and calcium binding protein or peptide labeling were included in this study. These stainings were also carried out separately and no change in the number of immuno-labeled neurons was observed. An example of a PV-immunoperoxidase staining is represented in Figure 1B.

**Table 1:** **Primary antibody list**.

**Antibody**	**Immunogen**	**Manufacturer/Investigator**	**Species**	**Catalog/lot number**	**Dilutions**
Parvalbumin	Purified frog muscle parvalbumin	Sigma	Mouse	P3088 Clone PARV-9	1:10000
				Lot #048K4752	
CCK	Synthetic human gastrin/CCK 2-17 conjugated with carbodiimide to keyhole limpet hemocyanin	Cure digestive diseases research center, UCLA	Mouse	#9303	1:10000
Calretinin	Recombinant rat calretinin	Millipore	Rabbit	AB5054/LV1532272	1:10000
				Lot #2049207	
Calbindin	Recombinant chick CaBP	Gift from Dr K. Baimbridge (2000)	Rabbit	R9501	1:10000
Reelin	Recombinant reelin amino acids 164-496	Millipore	Mouse	MAB5364	1:10000
				Lot #LV1791323	
Somatostatin	Somatostatin-14 conjugated to keyhole limpet hemocyanin	Gift from Dr A. Duchan (2000)	Mouse		1:7000
NPY	Neuropeptide Y coupled with bovine thyroglobulin with glutaraldehyde	Immunostar	Rabbit	22940 Lot #1112001	1:10000
VIP	Synthetic porcine VIP conjugated to bovine thryoglobulin with carbodiimide linker	Immunostar	Rabbit	20077 Lot #1129001	1:10000

### Antibody specificity

Frog muscle parvalbumin was used to produce the mouse monoclonal parvalbumin antibody (Clone PARV-9). This antibody reacts with human, feline, bovine, rat, frog, goat, fish, pig, canine, rabbit. Studies performed by the manufacturer indicate that this antibody recognizes parvalbumin in a calcium ion-dependent manner and does not react with other members of the EF-hand family, such as calmodulin, intestinal calcium-binding protein, S100A2 (S100L), S100A6 (calcyclin), the α and β chains of S-100. The specificity of this antibody was also tested on tissue from parvalbumin knockout mice and control mice and no staining was observed in mice lacking parvalbumin (Burette et al., 2009).

The mouse monoclonal CCK antibody (CURE, UCLA) was characterized using ELISA and radioimmunoassay (Ohning et al., 1994). This antibody specifically binds to the C terminal of CCK (Ohning et al., 1996). It has been widely used to label neurons in rodent brain tissue (Pawelzik et al., 2002; Klausberger et al., 2005).

Recombinant rat calretinin was used for the production of the polyclonal anti-calretinin antiserum in rabbit. According to the manufacturer's specifications, this antibody recognizes both calcium-bound and calcium-unbound forms of calretinin in immunoblots of rat tissues (manufacturer's specifications). In Western blots, this antibody labeled a single band at 29 kDa corresponding to the molecular mass of calretinin (Choi et al., 2010). This antibody reacts with human and rat.

The rabbit anti-chick recombinant CaBP, kind gift from Dr K. Baimbridge, University of British Columbia, detects calbindin immunoreactivity in nervous tissue of rodents (Baimbridge and Miller, 1982). It reacts with all mammalian species so far examined, including monkey, baboon and man. This antibody also detects CaBP in pigeon retina and chick brain and gut. It detects a single band of 28 Kd in both one and two dimension gel electrophoresis immunoblots.

The mouse monoclonal reelin antibody (clone G10) was prepared against the recombinant reelin residues 164–496 derived from mouse E15–E17 embryonic brain (De Bergeyck et al., 1998). On immunoblots from mouse and rat brain lysates, this antibody labeled a band at ~388 kDa that represents the reelin peptide. This antibody reacts with human, rat, mouse and rodent.

The mouse monoclonal antibody to somatostatin (kind gift from Dr A. Buchan, University of British Columbia) was raised against somatostatin-14 conjugated to keyhole limpet hemocyanin and was characterized using ELISA and immunocytochemical methods (Buchan et al., 1985). This antibody bounds to three forms: somatostatin-28 and both cyclic and linear forms of somatostatin-14. Preadsorption of this antibody with somatostatin-14 abolished all tissue staining and binding was unaffected by preadsorption with gastrin-17, motilin, and GIP.

NPY antibody was raised against Neuropeptide Y coupled to bovine thyroglobulin. All staining was blocked by preabsoprtion of the diluted antiserum with excess NPY according to the manufacturer data sheet. This antibody reacts with fish, frog, guinea pig, hamster, human, mouse, rat, squirrel and zebrafish.

The polyclonal antibody to VIP was raised in rabbit against porcine VIP conjugated to bovine thyroglobin with carbodiimide. In studies conducted by the manufacturer, preadsorption of the antibody with VIP (10^−5^ M) abolished all tissue staining. Pre-adsorption with the following peptides: Secretin, gastric inhibitory polypeptide, somatostatin, glucagon, insulin, ACTH, gastrin 34, FMRF-amide, rat GHRF, human GHRF, peptide histidine isoleucine 27, rat pancreatic polypeptide, motilin, peptide YY, substance P, neuropeptide Y, and CGRP did not affect the immunstaining. This antibody reacts with buffalo, chicken, guinea pig, hamster, human, monkey, mouse, rat, pig, sparrow, stingray, zebrafish.

### Determination of the borders of the CA2 region

The borders of CA2 were defined by superimposing immunoperoxidase staining of each of the calcium binding proteins, peptides and proteins with a Purkinje Cell Protein 4 (PCP4) staining (Figure 1D). The PCP4 staining was similar to the α actinin 2 labeling as shown in previous studies (Mercer et al., 2007) and in Figure 1C. Animals were perfused transcardially with a fixative solution (4% paraformaldehyde). Brains were removed and placed in a fixative solution (4% paraformaldehyde, 0.2% saturated picric acid solution, 0.025% glutaraldehyde solution in 0.1 M Phosphate buffer) for an hour. Brains were then washed. 50 μm coronal sections were cut, cryoprotected, incubated in 1% Sodium Borohydride for 30 min and in 10% NGS. Sections were then incubated overnight at 4°C in a primary antibody solution (Mouse anti-α-actinin, clone EA-53, Sigma, 1:1000 and rabbit anti-PCP4, HPA005792; Sigma, 1:200). Sections were then incubated in a secondary antibody solution, anti-mouse fluorescein isothiocyanate (FITC) and anti-rabbit Texas Red, made up in PBS. Sections were mounted on slides in Vectashield (Vector Laboratories) and studied by fluorescence microscopy as shown in Figure 1C. An example of the superimposition of the immunoperoxidase or fluorescence labeling and PCP4 staining is shown in Figure 1D.

### Analysis

For quantification in the hippocampus, the number of all GAD-67-, PV-, CCK-, Reelin-, VIP, SOM-, CB-, NPY-immuno-positive somata in CA1, CA2, and CA3 were determined using a DMR microscope (Leica Microsystems) in 5 adjacent coronal 50 μm-thick sections from at least 6 different rats per antibody. Slices used in this study were taken from the dorsal hippocampus in which the width of the CA2 region was on average 405 ± 35 μm to allow comparison of antibodies. The contours of all stained cell bodies in each section were drawn using a drawing tube and then digitized and counted using ImageJ software. The number of immunopositive interneurons was expressed as a percentage of GAD-positive cells in each layer of each of the three CA regions, SO, SP, SR, SL, and SLM. Neuronal density units were also analyzed for each marker in all hippocampal regions and layers using ImageJ software. Statistical analyses were performed using Mann Whitney U test (MW U) for a distinct comparison of the three CA regions. All data are represented as mean ± standard deviation (SD). All antibodies used in this study resulted in a clear staining of immunopositive cell bodies. Some antibodies (VIP, NPY, SS, reelin) only stained somata and/or very proximal dendrites, others (PV, CB, CR, CCK) also revealed dendritic and axonal staining. Partial reconstructions were only carried out using a drawing tube (x100 objective) when good antibody staining labeled interneuron dendrites.

## Results

### Parvalbumin-immunopositive interneurons in the hippocampal CA2 region

**Figure 2:**
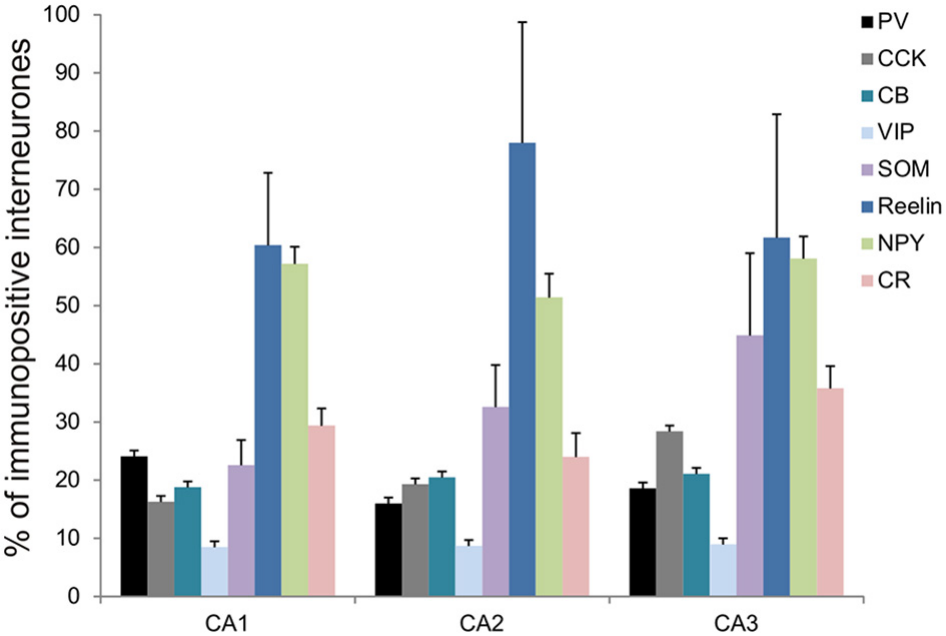
**Molecular characterization of interneurons in the CA2 region of the hippocampus**. Percentage of inhibitory cells that express parvalbumin (PV-black), cholecystokinin (CCK-gray), calbindin (CB-turquoise), Vasoactive Intestinal Peptide (VIP-light blue), somatostatin (SOM-purple), Reelin (dark blue), Neuropeptide Y (NPY-green), and calretinin (CR-pink) in each of the CA regions, CA1, CA2, and CA3. Total numbers of inhibitory neurons were obtained from the same sections labeled with anti-GAD67 antibodies.

**Figure 3:**
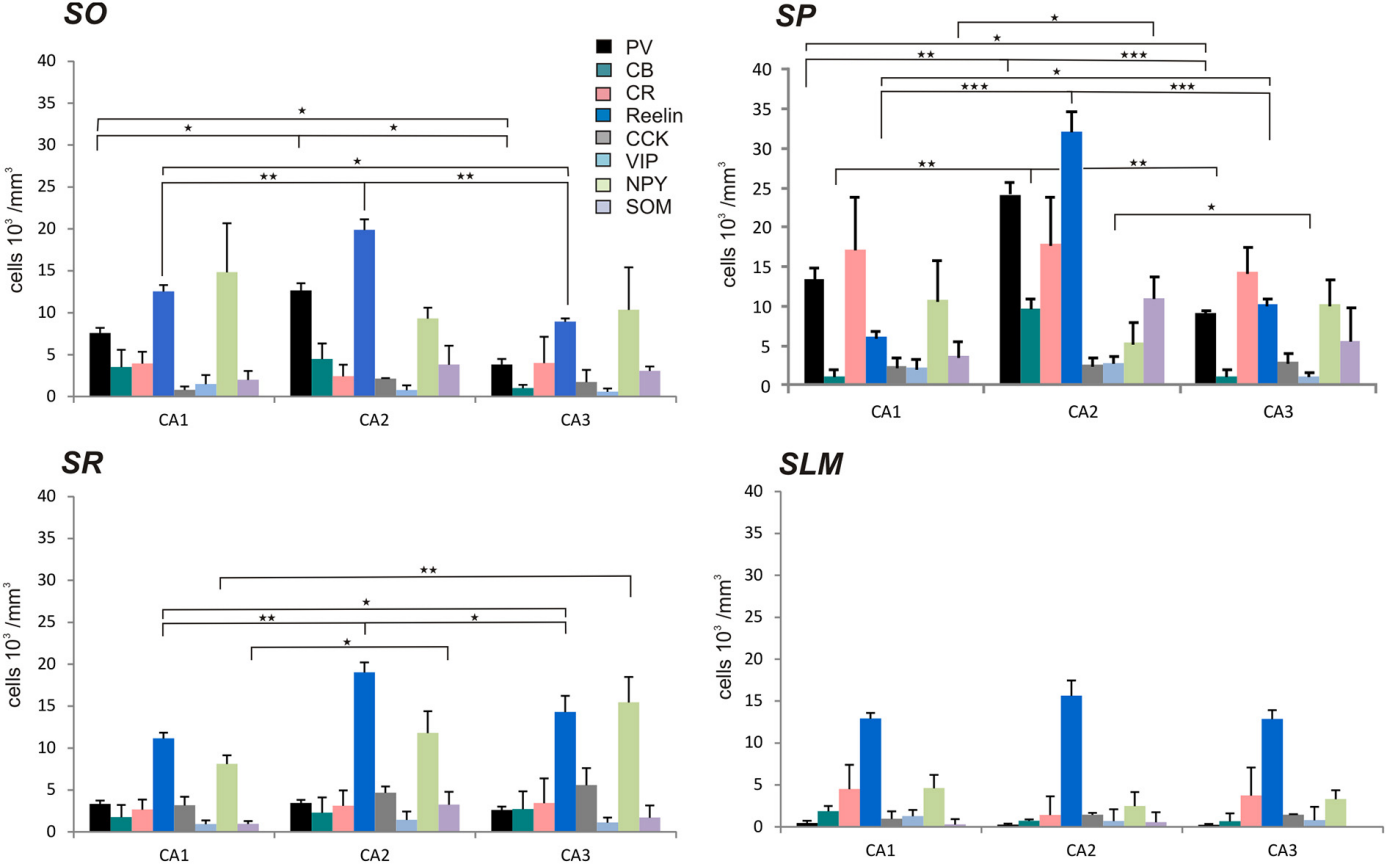
**Densities of immunolabeled interneurons in all layers of the CA1, CA2, and CA3 regions**. Cell densities of inhibitory cells (number of cell bodies per volume) that express parvalbumin (PV-black), calbindin (CB-turquoise), calretinin (CR-pink), Reelin (dark blue), cholecystokinin (CCK-gray), Vasoactive Intestinal Peptide (VIP-light blue), Neuropeptide Y (NPY-green), and somatostatin (SOM-purple) in *stratum oriens* (SO), *stratum pyramidale* (SP), *Stratum radiatum* (SR), and *stratum lacunosum moleculare* (SLM) of CA1, CA2, and CA3. There was a higher density of PV- and Reelin-positive neurons in SO in CA2 than in CA1 and CA3 (MWU *P* < 0.05). SP of the CA2 region contained a higher density of PV-, Reelin- and CB-positive interneurons than in CA1 and CA3 (MWU *P* < 0.05). There were also a higher number of VIP-positive somata in SP in CA2 than in CA3 and a larger density of SOM-positive cells in SP in CA2 than in CA1 (MWU *P* < 0.05). A higher density of Reelin- and SOM-positive neurons was found in SR in CA2 than in CA1 (MWU *P* < 0.05). ^*^P = 0.01 to 0.05; ^**^P = 0.001 to 0.01; ^***^P < 0.001.

**Figure 4:**
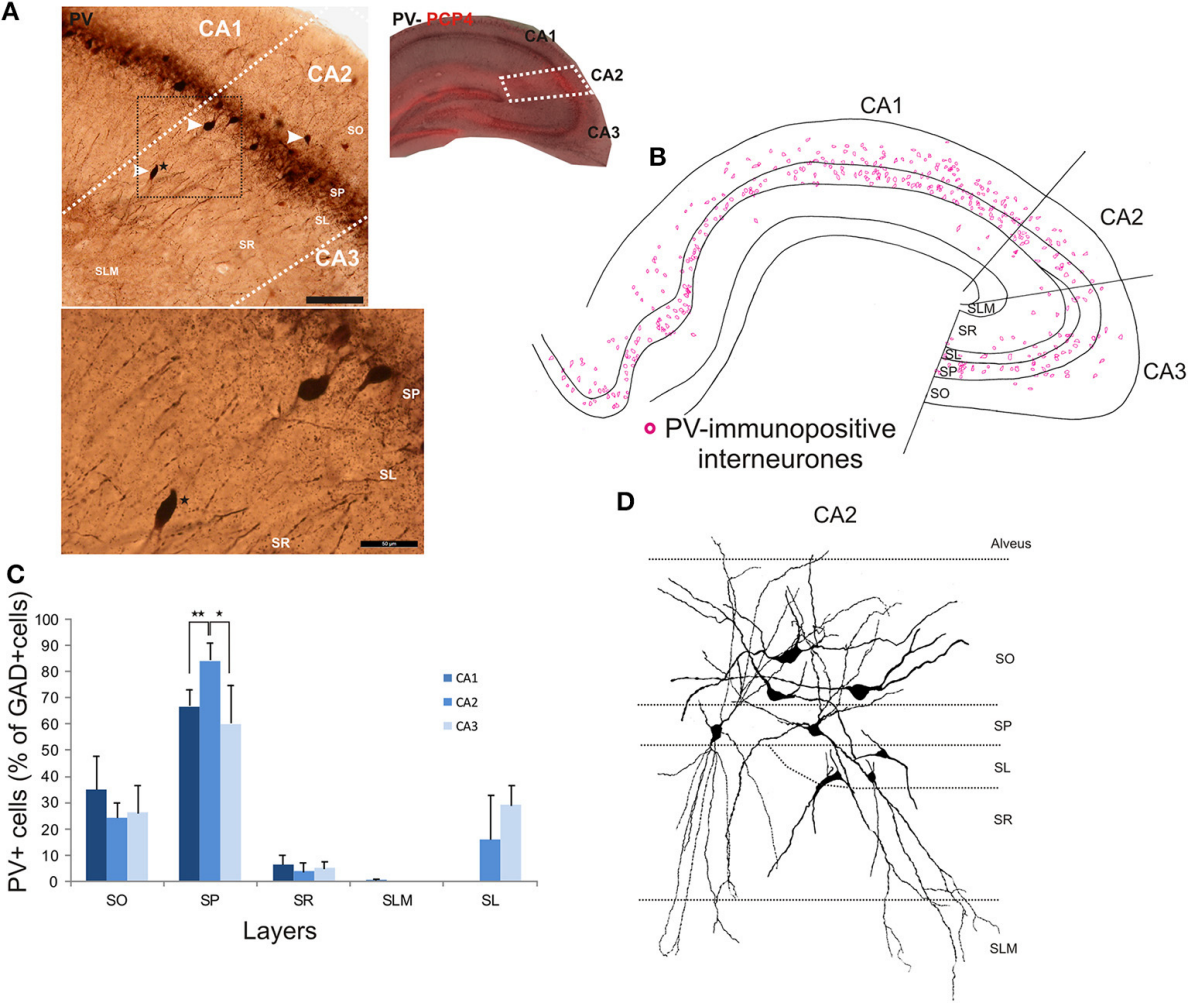
**Distribution of parvalbumin (PV)-immunopositive interneurons in CA2. (A)** Example of immunoperoxidase staining for PV. The CA2 region was delineated by superimposing the PV staining with the PCP4 staining (right panel) and represented with white dotted lines. Some immunolabeled interneurons in CA2 are indicated with white arrows. Note that the darker staining in SP represents axonal arbors of PV-positive cells. Staining (black dotted square) is represented at a higher magnification in the bottom panel. A PV-immunopositive interneuron (black star in both panels) was located in SR of the CA2 region. Scale bars 200 μm (top panel) and 50 μm (bottom panel). SO, Stratum Oriens; SP, stratum pyramidale; SR, stratum Radiatum; SLM, Stratum Lacunosum Moleculare; SL, Stratum Lucidum. **(B)** Distribution of PV-immunopositive cell bodies in all layers of the three CA regions, CA1, CA2, and CA3 (superimposition of five 50 μm-thick sections). PV-positive somata were mainly located in SO and SP of CA2, with very few cells in SR and none in SLM. **(C)** Distribution of PV-positive cells expressed as percentage of GAD-immunopositive neurons in CA1, CA2, and CA3 (5 sections- *n* = 7). ^*^P = 0.01 to 0.05; ^**^P = 0.001 to 0.01. **(D)** Partial reconstructions of PV-positive cells in the CA2 region. Interneurons with somata located in SO had mostly horizontal dendrites. PV-immunopositive cells with cell bodies in SP had vertical aspiny dendrites. However, some displayed horizontal dendrites. A few cells in SL were PV-immunopositive. Partial reconstructions of these cells revealed that their dendrites branched close to the soma and extended into SP, SR, and SLM.

The distributions of PV- immunopositive interneurons in CA2 were studied and compared with those of interneurons in CA1 and CA3. PV-immunopositive interneurons represented 16 ± 5.2% of the total interneuronal population in CA2, 24.1 ± 8.9% in CA1 and 18.6 ± 6.2% in CA3 (Figure 2). Neuronal densities of immuno-positive cells in SO, SP, SR and SLM of the three CA regions are plotted in Figure 3 and examples of the staining and distribution of PV-positive cells are represented in Figures 4A,B. Like CA1 and CA3, the somata of these interneurons in CA2 were primarily located in SO and SP, with very few cells in SR, or in SL and none in SLM (Figures 4B,C). However, PV-immunopositive somata represented a larger proportion of GAD-immunopositive cells in SP in CA2 than in CA1 or CA3 (MW U, *P* < 0.05, U_CA1_ = 0, U_CA3_ = 2) (Figure 4C), with a density of 24.1 10^3^ ± 1.6 10^3^ PV-positive cells/mm^3^ in CA2 SP compared with 13.3 10^3^ ± 1.5 10^3^ cells/mm^3^ in CA1 SP and 9 10^3^ ± 0.3 10^3^ cells/mm^3^ in CA3 SP (Figure 3). The majority of PV-immunopositive CA2 interneurons with somata located in SO had horizontal dendrites (Figure 4D). PV-positive interneurons with cell bodies in SP had dendrites that extended through SO and through SR and into SLM. Some had vertical dendrites confined to CA2, others had horizontal dendrites in SO that extended into CA1 and CA3. Partial reconstructions of PV-positive cells in SL of CA2 revealed that their dendrites extended into SP and SR of the CA2 region.

### CCK-immunopositive interneurons in CA2

**Figure 5:**
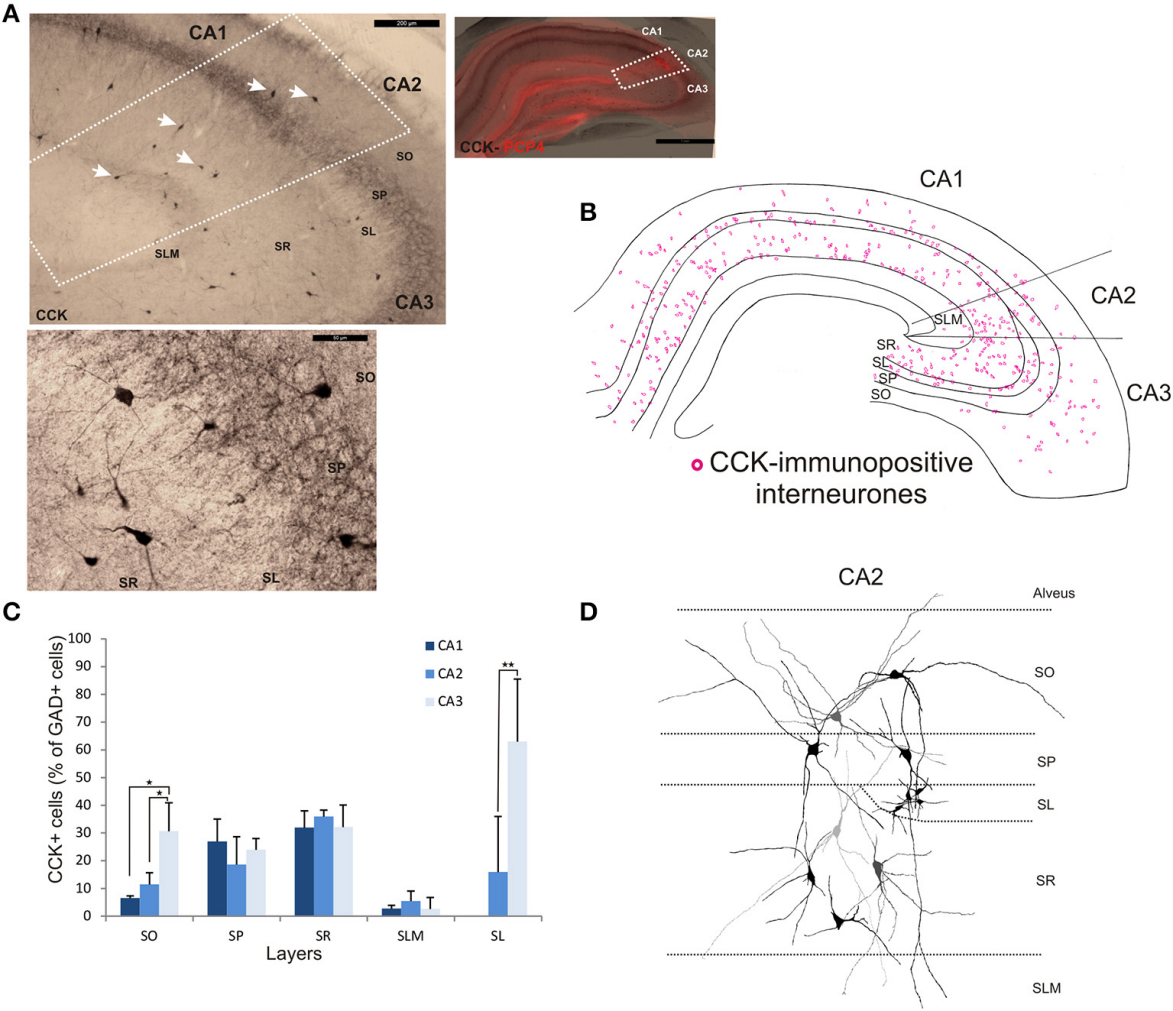
**Distribution of cholecystokinin (CCK)-immunopositive interneurons in CA2. (A)** CCK-immunoperoxidase staining. The borders of CA2 (white dotted lines) were defined by superimposing the CCK staining with the PCP4 labeling (right panel). Some of the CCK-immunopositive somata are indicated with white arrows. Example of CCK-immunoperoxidase staining in SP and SR of CA2 is shown in the bottom panel. Scale bars 1 mm (right panel), 200 μm (top left panel), and 50 μm (bottom left panel). SO, Stratum Oriens; SP, stratum pyramidale; SR, stratum Radiatum; SLM, Stratum Lacunosum Moleculare; SL, Stratum Lucidum. **(B)** Distribution of CCK-immunopositive neurons in all layers of the three CA regions, CA1, CA2, and CA3 (superimposition of five 50 μm-thick sections). **(C)** Number of CCK-positive cells expressed as percentage of GAD67-immunopositive neurons in CA1, CA2, and CA3 (5 sections- *n* = 7). As in CA1 and CA3, CCK-positive somata were located in all layers of the CA2 region, with the largest population in SR. Very few CCK-positive cell bodies were found in SLM in any region. ^*^P = 0.01 to 0.05; ^**^P = 0.001 to 0.01. **(D)** Partial reconstructions of CCK-immunopositive neurons in CA2. Note the small immune-reactive somata in SL.

The staining and distribution of CCK-immunolabeled neurons are presented in Figure 5. CCK-immunopositive interneurons represented 19.3 ± 4.4% of all interneurons in CA2, compared with 16.3 ± 3.4% in CA1 and 28.4 ± 10.5% in CA3 (Figure 2). CCK-immunopositive somata were mostly located in SR of CA2, with some cells in SO, SP, and SL (Figures 3, 5B,C). CA2 SLM contained very few CCK-positive somata. Comparing CA regions, CA3 contained the largest proportion of GAD-positive cells that were CCK-immunopositive in SO (MW U, *P* < 0.05; U_CA1_ = 1, U_CA2_ = 1) (Figure 5C) and a higher proportion of GAD- and CCK-positive somata in SL than in CA2 (MW U, *P* < 0.05; *U* = 0). CA1 is not considered to contain an equivalent layer. Partial reconstructions of the dendritic trees of CCK-immunopositive interneurons in CA2 revealed that those with somata located in SP had vertical dendrites that extended from SO to SLM, while those with somata in SO had predominantly horizontal dendrites (Figure 5D). In this, they resemble CCK-immunopositive cells in CA1. CCK-positive interneurons in SR of CA2 had dendrites that extended into SP and/or SLM. CCK-immunopositive somata in SL of CA2 appeared smaller than those in other layers. Their primary dendrites could be seen to branch close to the soma, but their weak dendritic staining did not allow more complete reconstructions.

### Reelin-immunopositive interneurons in CA2

**Figure 6:**
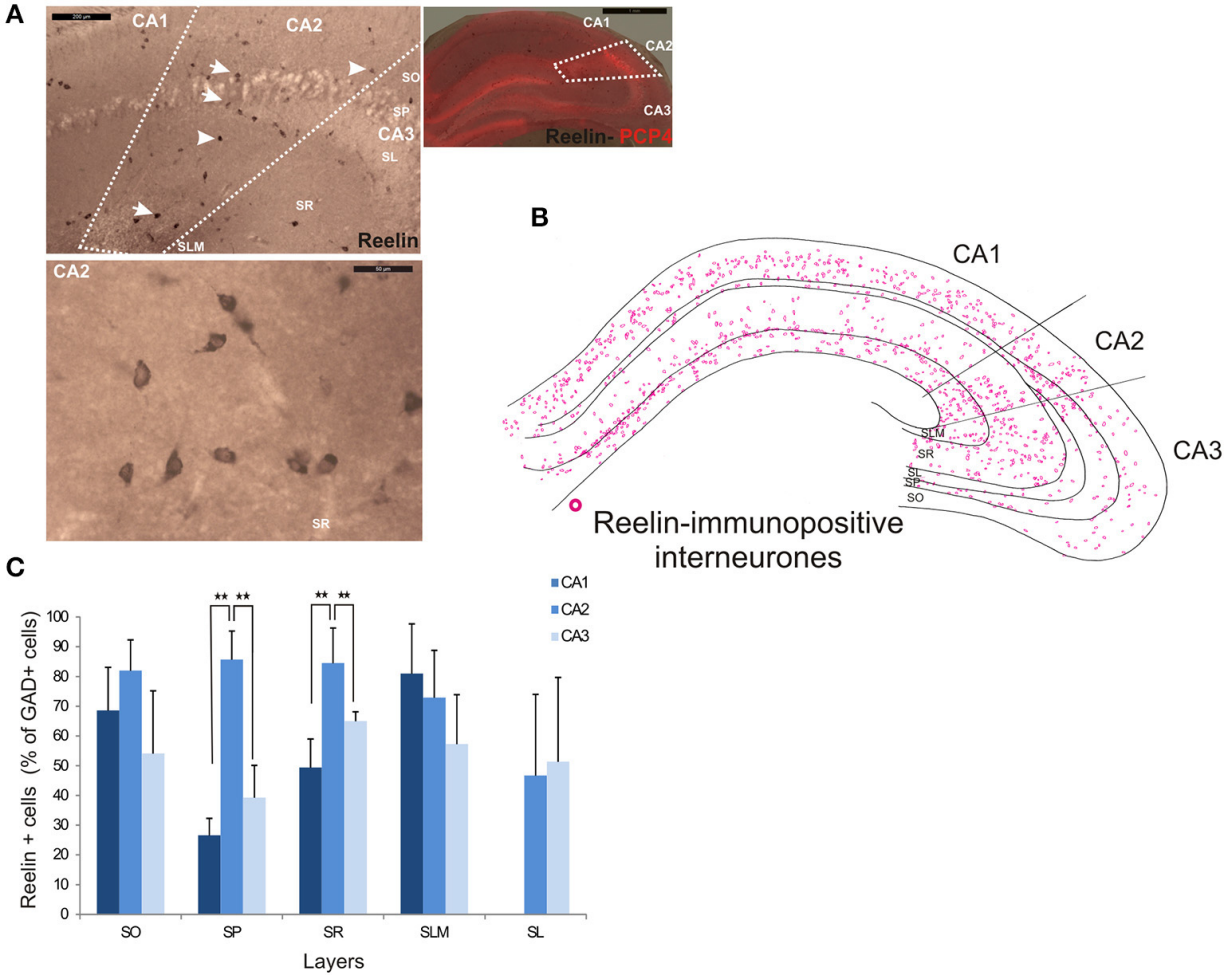
**Distribution of Reelin-immunopositive interneurons in CA2. (A)** Immunoperoxidase staining for Reelin. The borders of the CA2 region was defined by superimposing the reelin staining and the staining for PCP4 (right panel) and represented with dotted lines. Some reelin-immunopositive cell bodies are indicated with white arrows on the top left panel. Example of the reelin immunoperoxidase staining in SR of the CA2 region is shown in the bottom left panel. Scale bars 1 mm (right panel), 200 μm (top left panel), and 50 μm (bottom left panel). SO, Stratum Oriens; SP, stratum pyramidale; SR, stratum Radiatum; SLM, Stratum Lacunosum Moleculare; SL, Stratum Lucidum. **(B)** Distribution of reelin-immunopositive somata in CA1, CA2, and CA3 (superimposition of five 50 μm-thick sections). **(C)** Distribution of reelin-positive neurons in all layers of CA1, CA2, and CA3. Numbers are expressed as percentage of GAD-immunopositive neurons (5 sections- *n* = 7). Reelin-positive somata were distributed in all layers of the three CA regions. However, there was a larger proportion of GAD-positive cells that were reelin-positive in SP and SR of the CA2 region than in CA1 and CA3 (MWU *P* < 0.05). Reconstructions are not shown because this antibody does not label dendrites adequately. ^**^P = 0.001 and 0.01.

Reelin-immunopositive interneurons represented the majority of interneurons in all three CA3 regions, with 78 ± 20.7% of the total interneuronal population in CA2, 60.4 ± 12.4% in CA1 and 61.7 ± 21.2% in CA3 (Figure 2). Reelin-immunoperoxidase staining and distributions are shown in Figures 6A,B. Reelin-immunolabeled cell bodies were located in all layers of the three CA regions (Figure 6C). Interestingly, a larger proportion of GAD-positive neurons in SP and SR (mostly at the SR/SLM border) of CA2 was Reelin-positive compared with CA1 and CA3 (MW U, *P* < 0.05, U_CA1_ = 0, U_CA2_ = 0), with a density of 32 10^3^ ± 2.6 10^3^ reelin-positive cells/mm^3^ and 19 10^3^ ± 1.2 10^3^ cells/mm^3^ in CA2 SP and SR respectively (Figure 3). The reelin antibody staining only labeled the somata of the interneurons, preventing reconstructions of the dendritic trees.

### Somatostatin-immunopositive interneurons in CA2

**Figure 7:**
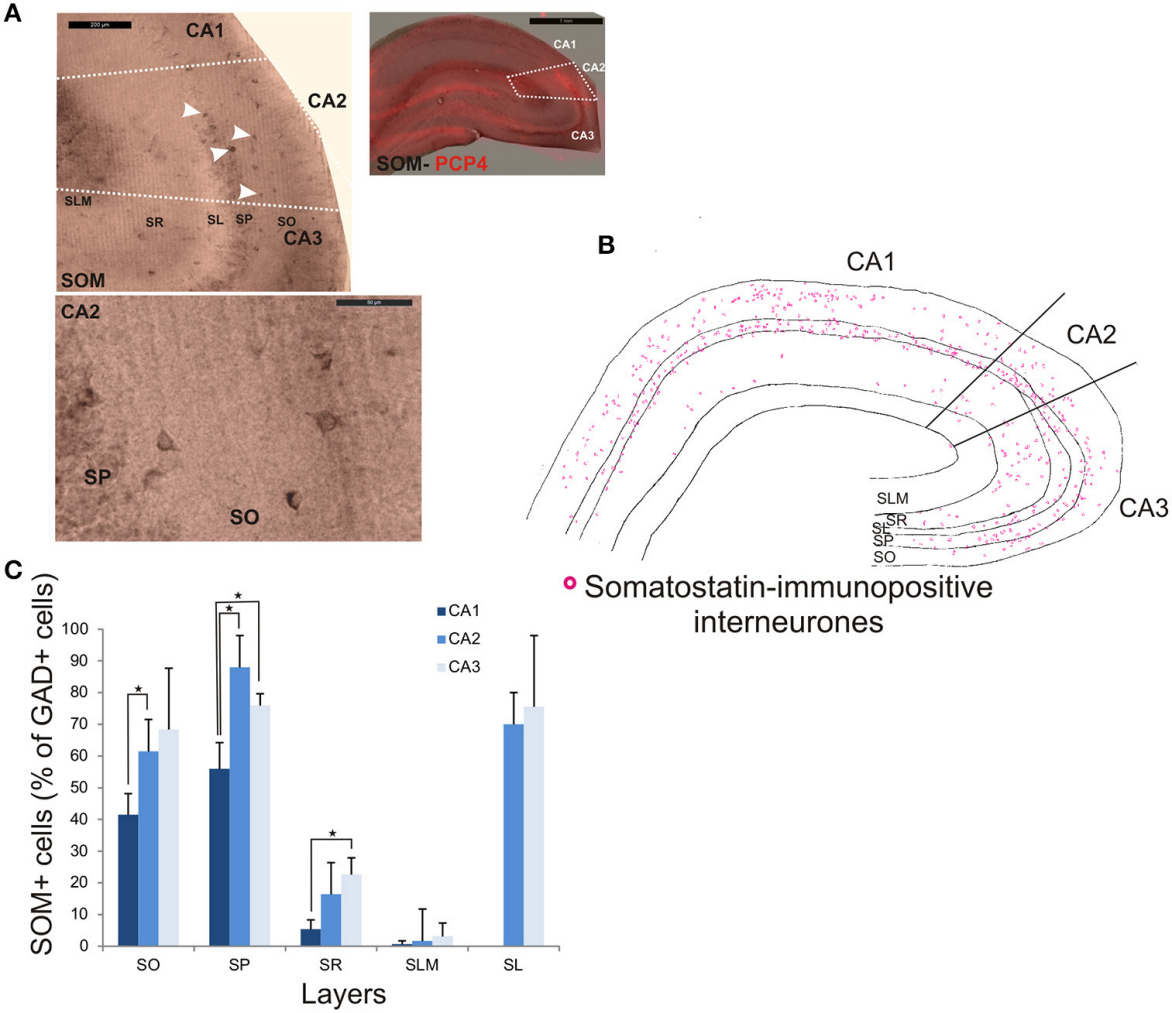
**Distribution of somatostatin-immunopositive interneurons in CA2. (A)** Example of immunoperoxidase staining for somatostatin. Superimposition of this staining with the labeling of PCP4 (bottom right panel) defined the borders of the CA2 region (white dotted lines). White arrows indicate some immunopositive somata (top left panel). SOM-immunopositive somata in SP and SO of the CA2 region are shown at high magnification in the bottom left panel. Scale bars 1 mm (top right panel), 200 and 50 μm (top and bottom left panels respectively). SO, Stratum Oriens; SP, stratum pyramidale; SR, stratum Radiatum; SLM, Stratum Lacunosum Moleculare; SL, Stratum Lucidum. **(B)** Distribution of SOM-immunopositive somata in CA1, CA2, and CA3 (superimposition of five 50 μm-thick sections). **(C)** Distribution of SOM-positive neurons in all layers of the three CA regions. Number of neurons was expressed as percentage of GAD-positive neurons (5 sections- *n* = 6). SOM-positive somata were mainly located in SO, SP, and SL of the three regions, with a few cells in SR and very few in SLM. There was fewer SOM-positive cell bodies in SO and SP of the CA1 region than of the CA2 and CA3 regions (MWU *P* < 0.05). SR of the CA3 region also contained fewer SOM-immunopositive somata than in CA1 (MWU *P* < 0.05). ^*^P = 0.01 to 0.05.

SOM-immunopositive interneurons represented 32.6 ± 7.2% of the total GAD-positive population in CA2, 22.6 ± 4.3% in CA1 and 44.9 ± 14.1% in CA3 (Figure 2). Examples of the staining and distribution of SOM-positive somata in all three CA regions are shown in Figure 7. SOM-immunopositive somata were mainly located in SO, SP and SL of the CA2 region, with a few cells in SR and very few in SLM (Figures 7B,C). The proportion of SOM-positive somata in SO and SP of CA2 was greater than in CA1 (MW U, *P* < 0.05, U_SO_ = 0, U_SP_ = 0) but similar to that in CA3 in all layers (MW U, *P* > 0.05, U_SO_ = 7, U_SP_ = 2, U_SR_ = 6, U_SLM_ = 5, U_SL_ = 6). Dendritic trees of SOM-positive interneurons were not labeled with the antibody used in this study.

### Calbindin-immunopositive interneurons in CA2

**Figure 8:**
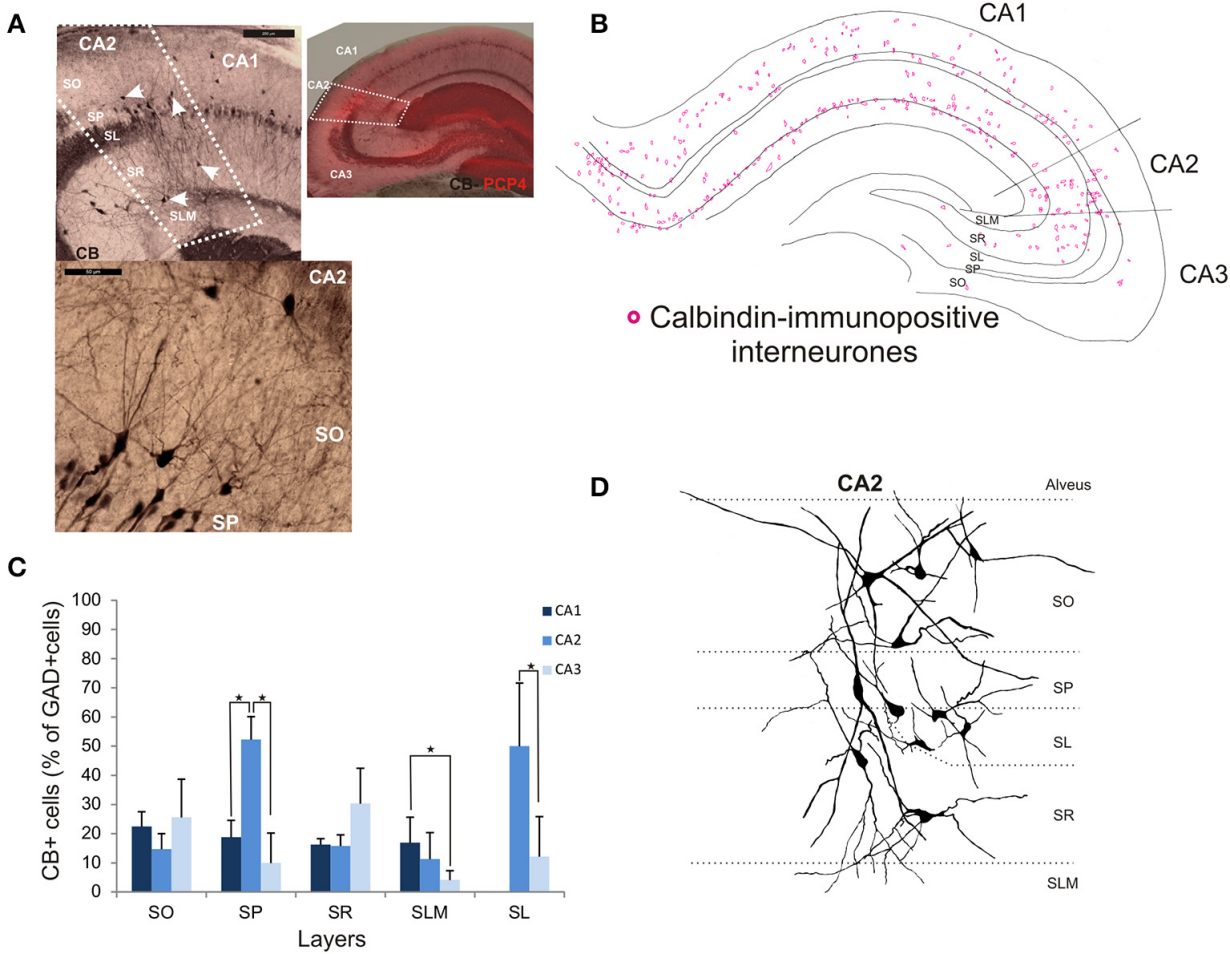
**Distribution of calbindin-immunopositive interneurons in CA2. (A)** Immunoperoxidase staining for Calbindin. The CA2 borders (white dotted lines) were determined by the superimposition of the CB staining and the PCP4 staining (right panel). Pyramidal cells, interneurons (white arrows) and mossy fibers expressed CB. Interneurons were characterized by their expression of GAD-67, their location and their dendritic pattern. Example of a calbindin immunoperoxidase staining in SO and SP of CA2 is shown in the bottom left panel. Scale bars 200 and 50 μm, top and bottom left panels respectively. SO, Stratum Oriens; SP, stratum pyramidale; SR, stratum Radiatum; SLM, Stratum Lacunosum Moleculare; SL, Stratum Lucidum. **(B)** Distribution of CB-immunopositive somata in CA1, CA2, and CA3 (superimposition of five 50 μm-thick sections). **(C)** Distribution of CB-positive neurons in all layers of the three CA regions [number expressed as percentage of GAD-positive neurons- 5 sections- *n* = 6)]. CB-immunopositive somata were present in all layers of CA2 with the largest number in SP and SL. In these layers, the proportion of interneurons immunopositive for CB was higher in CA2 than in CA1 and CA3 (MW U, *P* < 0.05, U_SP_ = 0, I_SL_ = 0). ^*^P = 0.01 to 0.05. **(D)** Partial reconstructions of CB-immunopositive interneurons in CA2. Interneurons with somata located in SO and SR presented horizontal dendrites. CA2 SP interneurons had dendrites that extended into SO and SR.

Calbindin immunochemistry yielded strong staining in pyramidal cells located at the SP/SR border of CA1 and CA2, in interneurons of all three CA3 regions and in mossy fibers (Figure 8A). CB-immunopositive interneurons were characterized according to their GAD 67-staining, the shape of their cell bodies and their dendritic morphology. They represented 20.5 ± 12.5% of the total interneuronal population in CA2, 18.8 ± 3.8% in CA1 and 21.1 ± 10.7% in CA3 (Figure 2). CB-immunopositive somata were present in all layers of CA2 (Figures 8B,C). However, a larger proportion of GAD-positive neurons in SP of CA2 was CB-positive compared with in CA1 or CA3 (MW U, *P* < 0.05, U_CA1, CA3_ = 0), with densities of 1.2 10^3^ ± 0.7 10^3^ CB-positive cells/mm^3^, 9.7 10^3^ ± 1.2 10^3^ cells/mm^3^ and 1.1 10^3^ ± 0.7 10^3^ cells/mm^3^ in SP of CA1, CA2, and CA3 respectively (Figure 3). SL of CA2 contained a larger number of GAD-positive cell bodies that were CB-immunopositive than SL of CA3 (MW U test, *P* < 0.05, *U* = 0). CB-immunopositive interneurons with somata located in SO of CA2 had horizontal dendrites that, in some cases, extended into CA3 and/or CA1 (Figure 8D), while CB-positive interneurons with cell bodies in SP had dendrites that extended vertically to SO and SLM. The similarity between these features of CB and of CCK immunopositive interneurons may result from co-expression of these two markers as seen in CA1. In addition, the cell bodies of CB-positive interneurons in SL, like those of CCK cells in SL, appeared to be smaller than those in other layers. Primary dendrites of these cells branched close to the soma and reached SP and SR of the CA2 region.

### VIP-immunopositive interneurons in CA2

**Figure 9:**
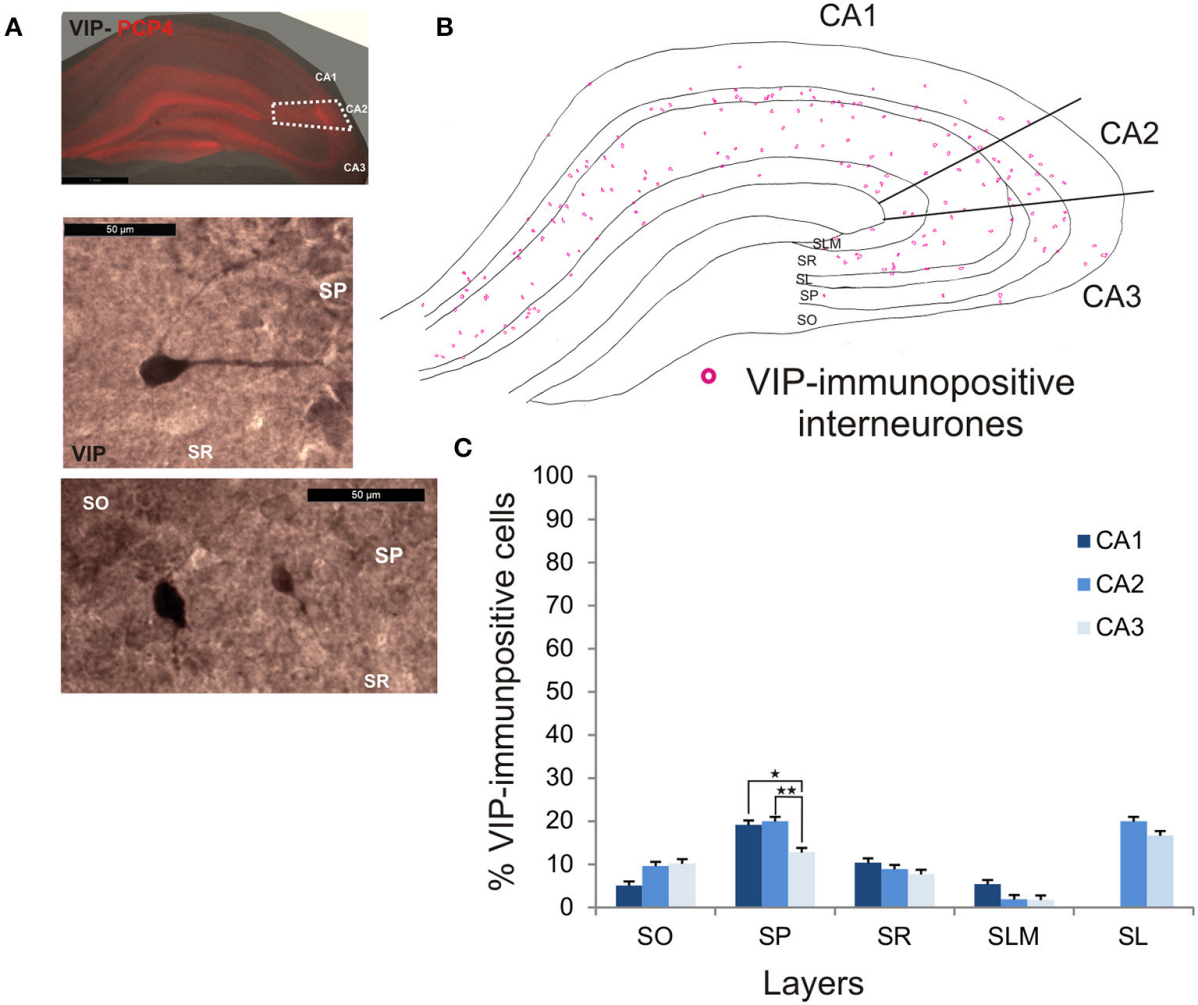
**Distribution of VIP-immunopositive interneurons in CA2. (A)** Example of immunoperoxidase staining for VIP. Superimposition of this staining with the labeling of PCP4 (middle panel) defined the borders of the CA2 region (dotted white lines). High magnification of VIP-immunolabeled somata in SO, SP, and SR are shown in the middle and bottom panels. Scale bars 1 mm (top panel) and 50 μm (middle and bottom panels). SO, Stratum Oriens; SP, stratum pyramidale; SR, stratum Radiatum; SLM, Stratum Lacunosum Moleculare; SL, Stratum Lucidum. **(B)** Example of the distribution of VIP-immunopositive somata in CA1, CA2, and CA3 (superimposition of five 50 μm-thick sections). **(C)** Distribution of VIP-positive neurons in all layers of the three CA regions (5 sections- *n* = 6). VIP interneurons constituted the smallest population in this study with a few VIP-immunopositive somata present in SO, SP, SL, and SLM and very few in SLM. There were more VIP-positive somata in SP of CA1 and CA2 than in CA3 (MW U, *P* < 0.05). ^*^P = 0.01 to 0.05; ^**^P = 0.001 to 0.01.

VIP-immunopositive interneurons represented 8.7 ± 4.7% of the total interneuronal population in CA2, 8.5 ± 3.8% in CA1 and 9 ± 3% in CA3 (Figure 2). VIP-immunopositive somata were mainly located in SP and SL of CA2 with a few cells in SO and SR (Figures 3, 9B,C). The number of VIP-positive cells in SP of CA2 was similar to that in SP of CA1 (MW U, *P* > 0.05, *U* = 12) but larger than that in SP of the CA3 region (MW U, *P* < 0.05, *U* = 0). VIP staining labeled only the soma of these interneurons preventing the reconstructions of the cells.

### NPY-immunopositive interneurons in CA2

**Figure 10:**
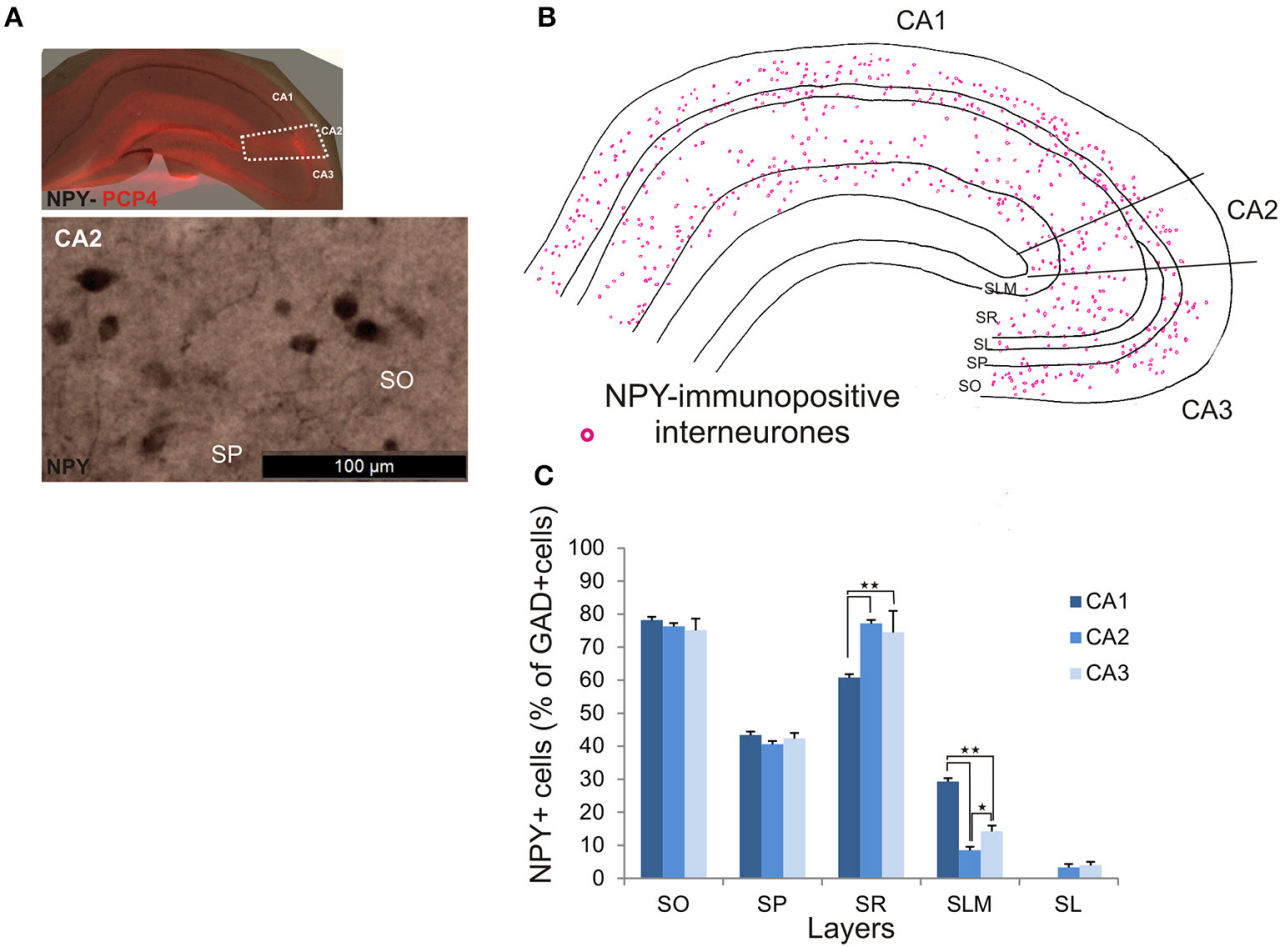
**Distribution of NPY-immunopositive interneurons in CA2. (A)** Example of immunoperoxidase staining for NPY. Superimposition of this staining with the PCP4 labeling (top panel) defined the borders of the CA2 region (white dotted lines). NPY-immunopositive cell bodies in SO and SP of the CA2 region are shown in the bottom panel. Scale bar 100 μm (bottom panel). SO, Stratum Oriens; SP, stratum pyramidale; SR, stratum Radiatum; SLM, Stratum Lacunosum Moleculare; SL, Stratum Lucidum. **(B)** Example of the distribution of NPY-immunopositive somata in CA1, CA2, and CA3 (superimposition of five 50 μm-thick sections). **(C)** Distribution of NPY-positive neurons in all layers of the three CA regions expressed as percentage of GAD-positive neurons (5 sections- *n* = 6). NPY-immunopositive somata were mainly located in SO, SP, and SR, with only a few cells in SLM and very few in SL. There were more NPY-positive somata in SR of CA2 and CA3 than in CA1 (MW U, *P* < 0.05). SLM of CA2 contained fewer NPY-positive cells than in CA1 or CA3 (MW U, *P* < 0.05). ^*^P = 0.01 to 0.05; ^**^P = 0.001 to 0.01.

NPY-immunopositive interneurons represented 51.4 ± 3.3% of the total interneuronal population in CA2, 57.2 ± 3.4% in CA1 and 58.1 ± 3.5% in CA3 (Figure 2). NPY-immunopositive somata were mainly located in SO, SP and SR of CA2 (Figures 3, 10B,C). There was a larger proportion of GAD-positive cells that were NPY-immunopositive in SR of CA2 than in CA1 (MW U, *P* < 0.05, *U* = 0). SLM of the CA1 and CA3 regions had a greater percentage of GAD- and NPY-positive cell bodies than CA2 (MW U, *P* < 0.05, *U* = 0).

### Calretinin-immunopositive interneurons in CA2

**Figure 11:**
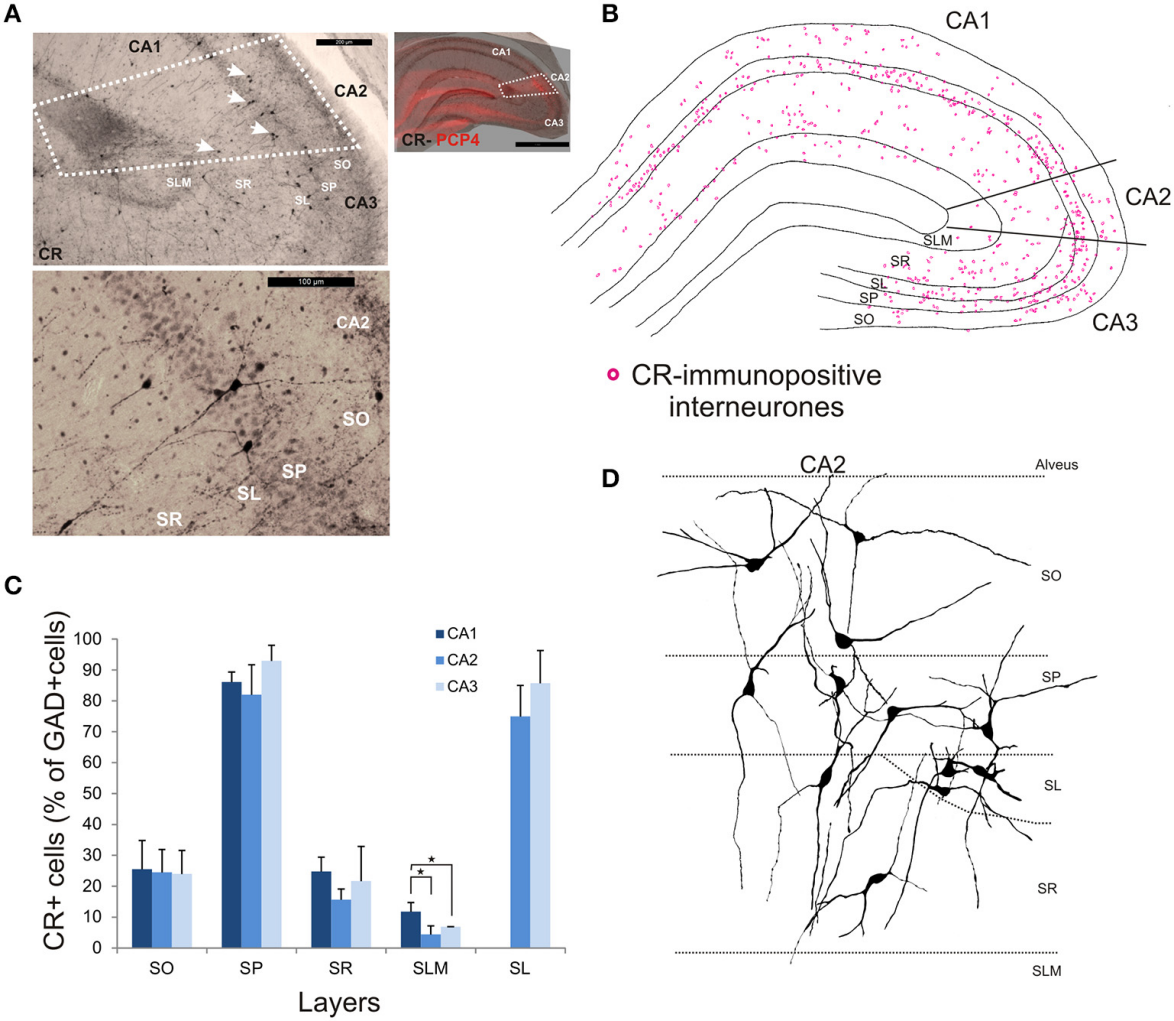
**Distribution of calretinin-immunopositive interneurons in CA2. (A)** Example of immunoperoxidase staining for CR. Superimposition of the immunperoxidase staining with the PCP4 labeling (top right panel) defined the borders of the CA2 region (white dotted lines). White arrows indicate some CR-immunopositive somata on the bottom panel. Example of the CR-immunoperoxidase staining in the CA2 region is shown in the bottom left panel. Scale bars 1 mm (right panel), 200 μm (top left panel), and 100 μm (bottom left panel). SO, Stratum Oriens; SP, stratum pyramidale; SR, stratum Radiatum; SLM, Stratum Lacunosum Moleculare; SL, Stratum Lucidum. **(B)** Example of the distribution of CR-immunopositive somata in CA1, CA2, and CA3 (superimposition of five 50 μm-thick sections). **(C)** Distribution of CR-positive neurons in all layers of the three CA regions expressed as percentage of GAD-positive neurons (5 sections- *n* = 6). More CR-immunopositive somata were located in SP and SL than in other layers. There were more CR-positive somata in SLM of CA1 than in CA2 or CA3 (MWU, *P* < 0.05). ^*^P = 0.01 to 0.05. **(D)** Partial reconstructions of CR-immunopositive interneurons in CA2. Most CR-positive interneurons with cell bodies located in SO had horizontal dendrites. Interneurons with somata located in SP had vertical dendrites that extended to SO and SR. However, some had horizontal dendrites that could extend into CA3. CR-positive interneurons in SL had dendrites that branched close to their somata and extended into SR of CA2 and, in some cases, to SR of CA3.

CR-immunopositive interneurons represented 24 ± 4.1% of the total interneuronal population in CA2, 29.4 ± 2.9% in CA1 and 35.8 ± 3.8% in CA3 (Figure 2). CR-positive somata were located mainly in SP and SL (Figures 3, 11B,C). The percentage of GAD-positive cells that were also CR-positive was similar in SO, SP, and SR of all CA regions (MW U, *P* > 0.05). There was, however, a larger proportion of GAD- and CR-positive somata in SLM of CA1 than in SLM of the CA2 and CA3 regions (MW U, *P* < 0.05, *U* = 0). Most CR-positive interneurons with cell bodies located in SO had horizontal dendrites (Figure 11D). Interneurons with somata located in SP had vertical dendrites that extended to SO and SR. However, some had horizontal dendrites that could extend into CA3. As seen with CB- and CCK-immunopositive cells, CR-positive interneurons in SL displayed smaller somata than those in other layers. Their dendrites branched close to their somata and extended into SR of CA2 and, in some cases, to SR of CA3.

## Discussion

The present study describes in detail the distributions of interneurons expressing widely used markers in the CA2 region for the first time and compare these distributions with those in the other CA regions. The pattern of distributions of the different markers in CA1 and CA3 presented in this study resembled that observed in previous studies (Kosaka et al., 1985; Sloviter and Nilaver, 1987; Sloviter, 1989; Freund and Buzsaki, 1996; Sloviter et al., 2001). The CA2 region contains interneurons that express, from the most abundant to the least abundant, Reelin, NPY, SOM, CR, CB, CCK, PV, and VIP (Figure 2) and their distribution in CA2 is layer-specific. Most distributions were similar to those in CA1 and CA3. However, GAD-positive cells are most likely to be PV-, reelin-, SOM-, CB-, and VIP-immunopositive interneurons in SP in CA2 than in other areas. SR of CA2 had a higher density of reelin- and NPY-positive cells than SR in CA1 and/or CA3, but fewer NPY- and CR-immunopositive cells were found in SLM in CA2 than in CA1. Although co-localization was not tested in this study, when the percentages of GAD-67 positive cells expressing each marker were added, percentages of 250, 237, and 277% in CA1, CA2, and CA3 respectively resulted, suggesting that, on average, each interneuron expresses 2–3 markers; the most likely combinations being PV with SOM including some that also express NPY, CB with CCK, reelin with NPY and SOM and VIP with CR, based on what is known in CA1 and cortex from previous studies (Rogers, 1992; Kawaguchi and Kubota, 1997; Alcantara et al., 1998; Pawelzik et al., 2002; Somogyi and Klausberger, 2005; Klausberger, 2009).

### Methodological considerations

Slices were taken from the adult rat intermediate hippocampus in which the CA2 region was of similar size (405 ± 35 μm) allowing the comparison of the staining for each antibody used in this study. We can, however, predict that the number of interneurons may vary depending on the species, gender and age of the animals. Immunoperoxidase staining was used to determine the density of different classes of interneurons. The protocol used (fixation, permeabilisation and visualization) was similar for each antibody. Although the dilutions were chosen for an optimal labeling of the cells, we may underestimate the numbers of stained neurons by using the same method for each marker.

In this study, interneuronal dendritic trees were only reconstructed when dendritic staining was adequate. Typically, as in CA1 in this and previous studies, the staining of calcium binding proteins labeled somata, dendrites and axons of interneurons whereas staining for other peptides or proteins resulted in poor labeling of dendrites with the exception only of CCK staining. These partial reconstructions did not therefore allow morphological comparisons of immunolabeled cells. To date, only PV-positive basket and bistratified cells and SP-SR cells have been fully characterized in CA2 (Mercer et al., 2007, 2012a,b). CCK-, CB-, and CR-immunopositive interneurons did not appear to be different from those previously described in CA1 and CA3, however, full morphological characterization of these cells has yet to be carried out.

### Large proportion of reelin-positive interneurons in CA2

Like other CA regions and indeed, like many other brain regions (Martinez-Cerdeno et al., 2002), the vast majority of interneurons in the CA2 region were immunopositive for reelin, with a larger proportion in SO and SP in CA2 than in CA1 and CA3. Reelin, a large secreted extracellular matrix protein, is essential for neuronal migration during development and for the correct organization of layered brain regions (D'Arcangelo et al., 1995; Tissir and Goffinet, 2003). A decrease in the expression of this protein may be involved in neurological diseases like epilepsy, Alzheimer's disease and schizophrenia (Guidotti et al., 2000; Heinrich et al., 2006; Haas and Frotscher, 2010; Kocherhans et al., 2010). Interestingly, these neurons were found in all layers of the CA2 region with a particularly high proportion of GAD67-positive cells in SLM being reelin immunopositive. There was also a larger number of reelin-positive somata in SO and SP of CA2 than in CA1 and CA3. Reelin-positive neurons have previously been shown to co-express somatostatin and NPY in adult hippocampus and cortex (Alcantara et al., 1998; Pesold et al., 1999). This may correlate with the large numbers of neurons in CA2 that were NPY- and SOM-immunopositive. Some reelin-immunopositive cells express calbindin and calretinin (Alcantara et al., 1998; Abrahám et al., 2004), but, reelin is rarely expressed in parvalbumin-, CCK-, or VIP-immunopositive interneurons (Alcantara et al., 1998; Miyoshi et al., 2010; Wierenga et al., 2010). From CA1 studies, the subtypes most likely to be reelin-positive may be back projection cells (Goldin et al., 2007) and hippocampo-septal cells (Toth and Freund, 1992; Gulyas et al., 2003) in SO, Ivy cells in SP and SR (Fuentealba et al., 2008) and neurogliaform cells at the SR/SLM border (Price et al., 2005). Conversely, OLM cells (Kosaka et al., 1988; Baude et al., 1993; Klausberger et al., 2003) in SO and Bistratified cells in SP (Buhl et al., 1996; Klausberger et al., 2004) may not be considered as probable reelin-expressing candidates as reelin is rarely expressed in parvalbumin-immunopositive interneurons in other CA regions (Alcantara et al., 1998; Miyoshi et al., 2010; Wierenga et al., 2010).

### Calcium binding protein- containing interneurons in CA2

Between 11 and 21% of interneurons of the CA2 region express parvalbumin and 20–25% calretinin. As in CA1, CA2 PV-interneurons are most likely to be classified as basket, bistratified or axo-axonic cells in SP and OLM cells in SO (Freund and Buzsaki, 1996; Pawelzik et al., 2002; Klausberger et al., 2003; Mercer et al., 2007). The two latter types have yet to be characterized in CA2, but are probably amongst those stained here for PV. There was a larger proportion of PV-immunopositive somata in SP of the CA2 region than in CA1 or CA3. Interestingly, these neurons and their connections are those most likely to be disrupted in epilepsy (Wittner et al., 2009). Calretinin-positive somata were mainly located in SO, SP, and SR of CA2. Their dendritic patterns resembled those in CA1 and these cells are most likely to be interneuron specific cells (IS-I or IS-II) (Acsady et al., 1996a,b; Gulyas et al., 1996).

### Peptide- and protein- containing interneurons in CA2

CCK-immunopositive interneurons of the CA2 region were mainly located in SP and SR with CCK-positive somata representing the largest population of neurons in SR. Dendritic patterns of CA2 CCK-positive cells were similar to those previously described in CA1, where CCK-positive interneurons include basket cells, Schaffer collateral-associated interneurons, apical dendrite innervating cells and perforant path-associated cells (Cope et al., 2002; Pawelzik et al., 2002; Klausberger et al., 2005; Somogyi and Klausberger, 2005; Klausberger, 2009). Although these cells have yet to be identified in CA2, they are the most obvious candidates for the CCK-positive interneurons described in this study.

VIP was the least abundant interneuronal marker in the CA2 region. VIP-immunopositive somata were mainly found in SO, SP, and SR of CA2. In CA1, most interneuron-specific interneurons are immunopositive for VIP (Acsady et al., 1996a,b). Whether this holds true for CA2 remains to be determined, but similar density and distribution in the two regions may indicate that this is the case.

### CA2 stratum lucidum

Although no thorny excrescences are present on CA2 pyramidal dendrites (Mercer et al., 2007; Kohara et al., 2014), mossy fibers have been shown to innervate pyramidal cells and interneurons in CA2 suggesting a role for this region in a distinct trisynaptic circuit, dentate gyrus-CA2-CA1 (Kohara et al., 2014). Mossy fibers appeared to target interneurons in SO, SP and at the border of SP and SL (Kohara et al., 2014). These interneurons, however, have yet to be classified. CA2 SL contained interneurons that expressed each of the markers tested, with the largest proportions being reelin-, CB-, SOM-, and/or CR-immunopositive. The location of somata close to a given input region does not always correlate with a strong innervation of those cells, but the positioning of the immunopositive small soma cells within the mossy fiber termination region suggests some functional correlate. Their dendrites branched close to their somata and extended into SP and SR, suggesting that they may receive input from all CA regions in addition to very proximal inputs from mossy fibers.

### Function of CA2 interneurons?

Growing evidence shows that the CA2 region, and more specifically inhibition in CA2, may play an important role in the hippocampal circuitry. CA2 interneurons target pyramidal cells locally, as well as in CA1 and CA3 and receive inputs from CA2, CA3, the supramamillary body, the amygdala, the entorhinal cortex and the dentate gyrus (Berretta et al., 2001; Bartesaghi et al., 2006; Chevaleyre and Siegelbaum, 2010; Ding et al., 2010; Piskorowski and Chevaleyre, 2011, 2013; Mercer et al., 2012a,b; Cui et al., 2013; Kohara et al., 2014). These interneurons are therefore ideally located to regulate the network activity and alteration of the inhibitory circuitry in CA2 appears to be linked to some neurological diseases. In human epileptic tissues, the lack of PV-immunopositivity in CA2 interneurons and a change in the expression of GABA_A_ receptor subunits appear to be responsible for the re-organization of connections in this region (Loup et al., 2000; Andrioli et al., 2007; Wittner et al., 2009). Similarly, a decrease in the number of CA2 interneurons, particularly PV-positive interneurons, has been associated with schizophrenia (Benes et al., 1998; Zhang and Reynolds, 2002; Nullmeier et al., 2011). Although some CA2 interneurons have been described in detail (Mercer et al., 2007, 2012a,b), very little is known about the different cell types in this region. The present study enables the start of their characterization. Determining where they are, how many they are, and the types of markers they express is an essential component of this characterization.

### Conflict of interest statement

The authors declare that the research was conducted in the absence of any commercial or financial relationships that could be construed as a potential conflict of interest.
